# A multi-perspective approach for defining neighbourhood units in the context of a study on health inequalities in the Quebec City region

**DOI:** 10.1186/1476-072X-6-27

**Published:** 2007-07-05

**Authors:** Alexandre Lebel, Robert Pampalon, Paul Y Villeneuve

**Affiliations:** 1 Centre de recherche en aménagement et développement, Université Laval, Québec, Canada; 2 Institut national de santé publique du Québec, Québec, Canada

## Abstract

**Background:**

Identification of socioeconomic and health inequalities at the local scale is facilitated by using relevant small geographical sectors. Although these places are routinely defined according to administrative boundaries on the basis of statistical criteria, it is important to carefully consider the way they are circumscribed as they can create spatial analysis problems and produce misleading information. This article introduces a new approach to defining neighbourhood units which is based on the integration of elements stemming from the socioeconomic situation of the area, its history, and how it is perceived by local key actors.

**Results:**

Using this set of geographical units shows important socioeconomic and health disparities at the local scale. These disparities can be seen, for example, in a 16-year difference in disability-free life expectancy at birth, and a $10,000-difference in average personal income between close neighbourhoods. The geographical units also facilitate information transfer to local stakeholders.

**Conclusion:**

The context of this study has made it possible to explore several relevant methodological issues related to the definition of neighbourhood units. This multi-perspective approach allows the combination of many different elements such as physical structures, historical and administrative boundaries, material and social deprivation of the population, and sense of belonging. Results made sense to local stakeholders and helped them to raise important issues to improve future developments.

## Background

During recent years, we have seen a number of works on the role of the local environment, or neighbourhood, in public health [1-17]. Although many recognize the important role of neighbourhood, these studies are still confronted with a major difficulty related primarily to the notion of "area", namely, what is a neighbourhood? And how can it be made operational?

We were able to raise these questions thanks to the implementation of a research project on health inequalities in the Quebec City region (Canada). In this article, we describe the implementation and results of a multi-perspective approach used for defining spatial units in the context of health studies on a local scale. Our purpose is two-fold. First, from a methodological standpoint, we wish to show how it is possible to integrate different ways of defining neighbourhood units and produce significant information on health variations at a local level. Second, on the intervention side, we wish to provide local stakeholders with a say in defining such units and facilitate the exchange of knowledge between them and researchers, as this currently constitutes a major public health issue[18].

We will first review the concept of neighbourhood units and identify key elements that help to make it operational. We will then describe how our neighbourhood units were drawn through a multi-perspective approach. Results will follow showing the layout of these units as well as their demographic, socioeconomic and health profiles. In conclusion, we will see advantages and limitations of this exercise, and how these neighbourhood units could be used to study the general health of a population at the local scale. Indeed, we show how neighbourhood units allow us to identify great disparities and how this information could be easily transferred to local stakeholders.

### The Concept of Neighbourhood

The beginning of the 20th century saw the first description of the local community as being a natural agglomeration. In 1915, Park [19] described these groupings as the results of the competition for land use between various businesses and groups of populations existing without formal organization. A review of the scientific literature over time would reveal a much more complex field of thoughts surrounding this concept than simply the result of competition due to free market forces [20-25]. Moreover, concepts like locality, district, region, boroughs, or local community are very close to that of neighbourhood [26] without many differences between them being clearly established in many researches [27]. But what is a neighbourhood and why are there so many concepts with a similar meaning?

A neighbourhood is often considered to be a living area as well as a place of work and a family environment. One will find people interacting for utility (grocery stores, medical clinics, schools, recreational parks, *etc*.), support or mutual aid (exchanges of services), or for pure socialization (the need to create bonds between individuals). It is a space we learn to recognize by moving throughout it while carrying social and economic activities such as visiting friends and shopping. The built environment and its social organisation can become familiar and could contribute to one's identity. A neighbourhood can thus become a reflection of oneself, one's values, aspirations and socioeconomic conditions [24]. It can also be freely selected or determined by these same socioeconomic conditions. In short, a neighbourhood is a place characterized by a specific collection of spatially based features that can be found at a specific geographic scale.

Since the work of Drummond in 1913 [28] who proposed asset of spatial unit that makes the concept of neighbourhood operational, many efforts have been devoted to the definition of neighbourhood units in the scientific literature. From a recent review of this literature, we conclude that there are two main categories of elements that need to be considered when identifying a neighbourhood unit: the inner characteristics and the geographic scale.

**The inner characteristics **refer to everything that could be considered an important element to characterize a neighbourhood. Although many authors have reviewed the notion of neighbourhood [4,12,27,29-31] Galster [22] has provided the most complete listing of those elements, grouping them in ten groups: structural, infrastructural, demographic, class status, public services, environmental, proximity, political, social-interactive and sentimental characteristics. We agree with his general and integrative definition of a neighbourhood, which is "a bundle of spatially based attributes associated with a cluster of residences, sometime in conjunction with other land uses [22], p. 2112)". However, obviously no neighbourhood can be homogeneous with regard to all these elements. Instead, it is characterized by a specific combination of homogeneity and/or heterogeneity of a few or many elements that make a neighbourhood different from its surrounding. This is known as the neighbourhoods' idiosyncrasy. The numerous inner characteristics could explain the fact that there are many related concepts to neighbourhood, and thus the concept chosen depends on the point of view used to describe the neighbourhood. Kallus and Law-Yone[23] detailed some of these viewpoints. They explain that the concept of neighbourhood could be used in a humanistic, instrumental and phenomenological approach. The humanistic approach emphasizes social bonds in a physical setting. The instrumental approach sees the neighbourhood as a functional system used for planning purposes. The phenomenological approach considers, rather, bonds between places and people created by time and events, and produces a specific dynamic that influences organisation and architectural typologies. As an example, one could use the word community when taking a humanistic approach, whereas someone employing an instrumental approach might use the word district. All these points of view refer to some specific aspect of a territory's reality. We believe that this reality shall be best represented if they are all taken into account when one tries to define neighbourhood units.

**The geographic scale **is also an important aspect to consider when defining neighbourhoods units. Indeed, their relation to the territory and their principal characteristics might change with the scale. Based on Suttles' work [25], Kearns and Parkinson [24] determined the dominant function of each of three spatial levels of the concept of neighbourhood which are intrinsically connected inside the same area: the home area, the locality and the urban district. The home area refers to belonging and family, where the psycho-social purposes of neighbourhood tend to be strongest and it is typically defined by the area within a 5–10 minute walk around someone's residence. The locality refers to the wider area where residential activities are still highly predictable, familiar, and is visited frequently. The urban district refers to an even larger landscape of social and economic opportunities which might vary considerably from one individual to another. In this way, neighbourhoods can be seen as overlapping areas in relation to one's needs, the whole being centered on the residence. Moreover, the scale of a neighbourhood shall be very different between urban and rural areas, where notions like distance or local are different. Therefore, the concept of neighbourhood is not necessarily confined to urban milieus[32]; it could simply be another way to express the idiosyncrasy at a proper geographic scale. A rural neighbourhood, by example, could cross municipalities' frontiers because the social dynamic and public services sharing can be very high between two or three particular municipalities.

Beyond these considerations many problems remain: where does one draw the line? What inner characteristics are important? And what is a proper scale? Indeed, it is a time-honoured difficulty for geographers that consists in placing relevant limits around specific areas (P. Buache 1752; in [33]). How then is it possible to make operational such a general and multidimensional concept around which there is no consensus?

### Neighbourhood Unit as an Analysis Tool

Recent works emphasize the importance of the method used to define neighbourhood units. This is of the utmost concern given the effect this definition can have on the study. The use of borders established more or less arbitrarily can generate serious information biases and reduce the validity of analyses. Most approaches adopted to establish such units are of a statistical nature [34] or call upon geographical borders defined for policy purposes [35]. These boundaries often have only one rationalization: quick access to available information. It is well-known that these approaches remain largely incomplete because they lack a conceptual framework [36]. As the neighbourhood integrates place as well as people [37], its conceptualisation must consider characteristics of both place and people, and the interaction between them. It must also consider that a neighbourhood is always a part of a larger whole [36]. Coombes [38] argued that "comparing areas without the confidence that they have been consistently defined creates problems for both in-depth local studies – where the definition of areas will intimately shape the findings – and also in comparative spatial analyses where the importance of study-area boundaries shaping the results has been termed the modifiable areal unit problem (Openshaw and Taylor, 1981)" (MAUP). Actually, this problem could constantly show up for as long as one works with boundaries. On the other hand, the advantages of using boundaries, meaning the possibility of comparing any set of data on the same geographical frame, or of presenting complex data in a simple way, are large enough to incite many researchers to work at reducing its effect as much as possible. In any case, a good conceptual framework of the studied territory is still needed.

Several authors have already considered certain alternatives to reduce this bias [4,27,30,34,38-42], by suggesting new approaches based on criteria such as inhabitants' perceptions, administrative borders, demography, economy landscape or on historical criteria. Nevertheless, these proposals are not free from limitations. For example, they give priority to only one perspective (generally socioeconomic) instead of using multidimensional viewpoints. Nor do they take into consideration the point of view of residents and local decisions makers. Generally, they leave little or no room for caregivers or other administrative actors in the definition of the local living area. Propositions for interventions may be made, but are likely to be short-lived if the local actors concerned do not recognize the local area or if it makes no sense to them. If one considers the MAUP to be the uncertainty about which geographical set of units to use for analysis [43], one will realize that the viewpoint of local stakeholders is of prime importance for knowledge transfer. Indeed, the personal knowledge and experience of local decision makers can help to overcome this uncertainty and even, in some cases, to assume it away.

Unfortunately, there is no magic formula that could bring together all relevant elements of a neighbourhood to create an all-purpose spatial grid. However, we believe that the two categories of elements described earlier, e.g. the inner characteristics and the scale, are particularly relevant for defining spatial units related to health, and that choices are to be made regarding those elements before defining neighbourhood units. What follows are the choices we made that led to the creation of geographical units that could be used to manage the multidimensional concept of neighbourhood in the context of a study on health inequalities.

## Methods

### Studied Areas

We conducted this study in three territories of the Quebec City region: the boroughs of both Saint-Louis and Banville, and the rural county (MRC) of Verdier; for ethical reasons, locality names are fictional. They were selected for their comparability on certain aspects and contrasts on others [44]. Furthermore, they represent three basic types of milieus, namely a central urban district, a suburban and a rural area.

In order to produce a set of neighbourhood units as close as possible to the way the territories are built, lived, and perceived, meaning where the neighbourhood's idiosyncrasy is best represented, a three-prong approach was developed combining and integrating historical, socioeconomic and perceptual viewpoints.

### The Historical Perspective

This perspective is based on locating all institutional, private or public demarcations used during the past forty years before the beginning of this study (from 1963 to 2002). The forty year period was considered since it is about the average duration of an adult's active life. The collected limits could be, for instance, from primary schools' catchment area, regional planning units, fire or police security dispatch zones, municipalities or parishes' boundaries. About 40 different maps have been collected for each studied territory. Every single limit of each map was weighted according to four criteria: the length of utilisation, the decade of use, the relevance of a limit according to the research theme (social and health inequalities) and the collected information's accuracy. For example, a numerical map of the parish boundaries surrounding small and well integrated communities (used for a social purpose), that are still in use after forty years, received a much better score than an old photocopy of the electoral sectors of 1965–1969 (used for an administrative purpose). All possible ways to subdivide the territory that could have been found were introduced in our historical database.

All maps were then geocoded and integrated in a geographical information system (GIS). A topological structure was made using street network for urban areas and the regional planning units for rural areas. Every segment of the topological structure was given a weight according to the four criteria. Natural breaks in the distribution of those weights served as class thresholds for defining the frequency of the limits use: very often, often, sometimes and never. Details of this method are described elsewhere [45]. In summary, it provides a synthesis of all maps that have been filed and used by various administrations during the last forty years, identifies the most frequently used borders during this period, and thus, the outcome could serve as a proxy of the locality's spatial frame of reference.

### The Socioeconomic Perspective

For the definition of neighbourhood units according to a socioeconomic perspective, we had access to a deprivation index developed by Pampalon and Raymond [46]. This index describes the material and social aspects of deprivation according to Statistics Canada's dissemination area (DA) (dissemination areas are the smallest geographical units available in Canada. The average population of a DA is about 600 persons). Material deprivation is mainly associated with education, employment and income, whereas social deprivation refers primarily to single parenting, marital status, and living alone. These indicators were selected for their known relations with a large number of health problems, their affinities with the material and social forms of deprivation and their availability by DA. Both dimensions of deprivation are in fact the two main components of a principal component analysis carried out on the above socioeconomic indicators.

In order to provide a unique statistical representation of deprivation for each territory, we carried out a cluster analysis by dissemination area for each aspect. We used the K-Means cluster analysis since the results generated are more compact than those obtained by the hierarchical method, exclude any possibility of overmatching and maximize the internal groupings' homogeneity [47]. Following preliminary analyses, we determined that five groups were adequate to spatially reveal the main deprivation differences. Each group brings together DAs with similar factor scores on both deprivation aspects.

The result of this approach is mainly cartographic, offering a portrait of the population's material and social conditions' internal diversity. It allows to locate places with similar levels of deprivation into five groups, from most privileged to most deprived, and the evaluation of the level of adjacency for each of the five groups.

### The Perception Perspective

For this perspective, a focus-group type exercise, chaired by the first author of the present study, was carried out with local key actors who have had an excellent knowledge of one of our three study areas. We chose to work with local key actors because they bring a valuable and coherent point of view of the territory since they can both look at it as a whole and give advice about its interactions with the region, and discuss specific details within it. We then selected candidates with the greatest professional or lay experience linked with the territory, while making sure that each one of them had a different expertise in order to diversify the discussions' points of view. In each territory, we held a workshop of three hours for local key actors coming from activity sectors such as municipalities' administration, community groups, school board, and community health and social services. Five to eight people per territory took part in this workshop. The goal was to collect their overall perception of the territory, to understand what a neighbourhood unit meant to them, to insure the integration of the three perspectives, and to circumscribe a set of neighbourhood units.

Before the exercise, all the participants were informed of the objectives and the context of the study as well as the work already carried out on their territory for the definition of neighbourhood units, e.g. the result of the historical and statistical perspectives. It was essential to provide this information at the beginning of the workshop in order to give a basis on which to work and on which participants could reach a consensus. We then asked the participants to map out, according to them, what would be their personal proposal of neighbourhood units on their territory by leaving them completely free to use any criteria they considered most significant. They could select or discard layouts suggested by the historical and statistical perspective and/or modify them according to other criteria they considered relevant.

Only two constraints of a statistical nature were established. The first constraint was that units must gather an integer number of dissemination areas; this criterion allowing units to be perfectly compatible with Statistics Canada's available census data and other databases. The second constraint was that the units should contain approximately 5,000 inhabitants (+/- 3,000) to carry out analyses about rare events (low birth-weight, death rate, *etc*.) with the minimum level needed for statistical significance, and to keep a local perspective. There is no gold standard number for neighbourhood unit analysis and the selected values are always more or less arbitrary. Let us say, for instance, that with an average death rate of 7 per 1,000 persons, our selected number should bring between 70 and 280 deaths for the five-year period considered, which seems adequate to detect significant statistical differences.

During this exercise, material such as pens, markers and a collection of detailed large scale maps (including the specific population in each DA) were given to each participant. They were asked to draw neighbourhood unit boundaries according to what they believed to be the most relevant criteria. Every participant was given equal time to present and justify his or her choice of neighbourhood units and then a two hour discussion followed in order to arrive at a consensus on a final set of units. During the discussion, the chairman insured that all participants could express his or her point of view and that the final set of neighbourhood units was satisfactory to all.

**Figure 1:**
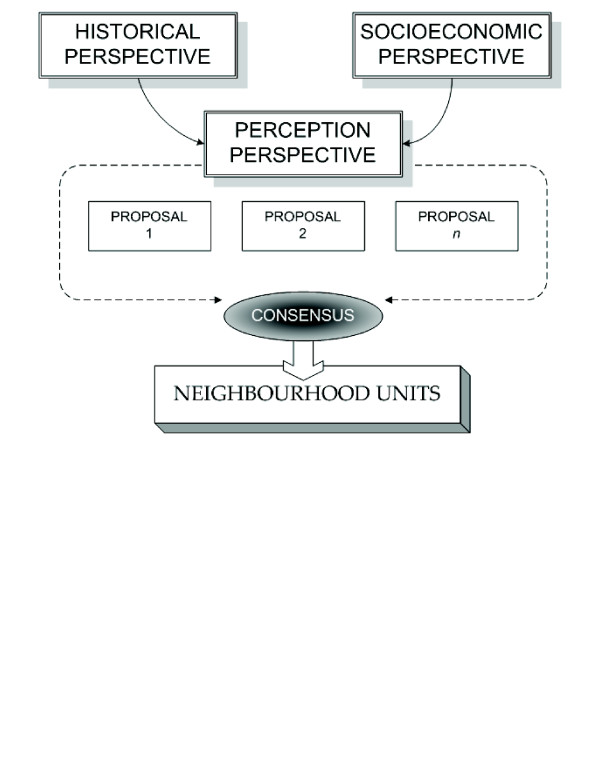
Integration process of the historical and socioeconomic perspectives by local professionals.

The historical perspective identified the most frequently used limits generated by public services, local policy or by urban infrastructures for its recent history. The socioeconomic perspective offered a picture of the current deprivation status while locating some spatial clusters. The perception perspective not only provided useful information on social interactions, sense of belonging, accessibility to various services, and on local characteristics, but also made it possible to integrate the whole procedure (figure 1). It is with this procedure that we obtained a final set of neighbourhood units among which we investigated health disparities at the local scale.

## Results

The main results of this research lie in three kinds of outcomes. We will first report on the neighbourhood units' boundaries drawn from the workshops held in each territory, mentioning what the most relevant elements used for the definition are. Second, we will briefly describe the neighbourhood units' profiles in terms of demographic, socioeconomic and health status characteristics, and then discuss the knowledge transfer process.

### Neighbourhood Units' Boundaries

#### Saint-Louis's neighbourhoods

**Figure 2:**
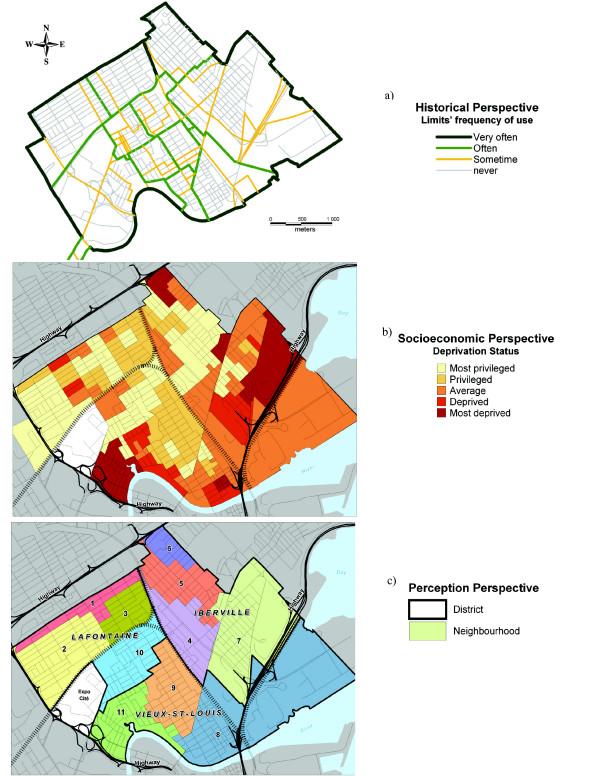
Saint-Louis' neighbourhood definition from the historical^a^, socioeconomic^b ^and perception^c ^perspectives.

Five people whose activities related to the area of Saint-Louis and who came from different professional environments (health services, credit union, borough's social development, school board, local center of development) took part in the workshop on defining neighbourhood units. They delineated 11 units while referring as much to the historical and statistical perspective as to their own perception of the territory, without necessarily using the same criteria for each unit (figure 2).

The historical perspective highlighted the frequency or the intensity with which some boundaries have been used during the last forty years (figure 2a). This analysis revealed that the old parish boundaries were, and still are, a significant spatial frame of reference even if those boundaries no longer exist. The socioeconomic perspective (figure 2b) allows an easy localization of the most privileged or deprived sectors, the evaluation of the borough's internal homogeneity, and the level of space adjacency of the five statistical groupings. Finally for the perception approach (figure 2c), the contribution of the local key actors was necessary to identify significant characteristics that could not be seized by the other two perspectives, and to sort out which features were relevant for defining Saint-Louis's neighbourhoods. This exercise revealed that the most important characteristics were: the socioeconomic situation, some physical barriers (rail way, commercial axes and major streets), the ancient parish boundaries, the strong sense of place, the presence of socially relevant institutions (schools, churches, cooperatives), dwelling types, the physical state of residences, and the ratio of tenants/homeowners.

We then combined these 11 neighbourhood units to approximate the spatial definition of local districts, which are administrative areas used by the City and the Borough councils for planning purposes in order to increase the relevance of this spatial grid for intervention. In Saint-Louis, three such districts exist, namely: Lafontaine, Iberville and Vieux-Saint-Louis (figure 2c).

#### Banville's neighbourhoods

In Banville, five people also coming from different professional environments (health services, city planning, local social development, leisure organization, local development center) took part in the workshop. They delineated 15 units using the three suggested perspectives.

**Figure 3:**
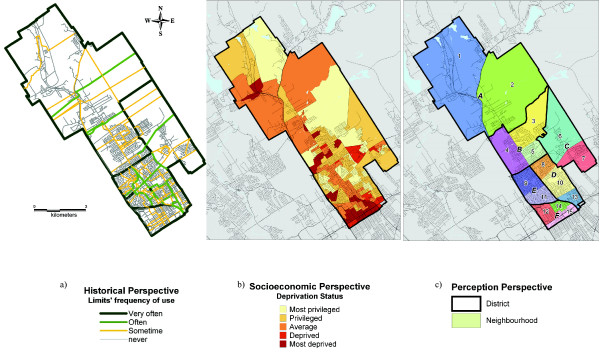
Banville's neighbourhood definition from the historical^a^, socioeconomic^b ^and perception^c ^perspectives.

Boundaries located by the historical perspective were less clear than those found in Saint-Louis (figure 3a). The fact that most of Banville's current built environment was developed under the supervision of four independent municipal administrations that were not coordinated and, at the same time, that the clergy had considerably lost its social and political influence, explains to some extent this lack of spatial frames of reference. Nevertheless, some significant boundaries could be brought out of this web, mainly previous municipal limits, some of the oldest parish, and some newly constructed areas. The socioeconomic perspective made it possible to bring out two specific elements of Banville (figure 3b): a good level of adjacency in some statistical groupings, in particular in the southern sector, and some deprived enclaves within a generally privileged sector. Consequently, Banville proved to be very homogeneous in some areas and heterogeneous in others.

Since the historical perspective offered confusing results and the socioeconomic approach revealed certain singularities, the assistance of the local actors was particularly useful in the case of Banville. Indeed, many other important elements were identified and located by them, like the socioeconomic situation, the types of housing (multifamily vs. one-family), the types of urban development, the social contacts, the presence of immigrant populations, the presence of some natural physical borders (forest, hills, *etc*.), some main roads and the old railway. All of these elements were considered as being of significant importance in partitioning Banville's neighbourhood units, and in helping to integrate information contained within the historical and socioeconomic approach. According to the actors, the final result (figure 3c) gives a representative portrait of the social reality of the borough.

Finally, we combined these 15 neighbourhoods to approximate the spatial definition of local districts, which have been recently created by the City and the Borough councils for planning purposes. In Banville, six such districts exist, but do not possess official names yet and are referred to a letter (units A to F, figure 3c).

#### Verdier's neighbourhoods

Eight people coming from different professional environments (health services, youth employment services, regional planning, co-operative association of family economy, local development agency, community local development agency, local centre of employment, school board) took part in the workshop and delineated eight units in Verdier.

**Figure 4:**
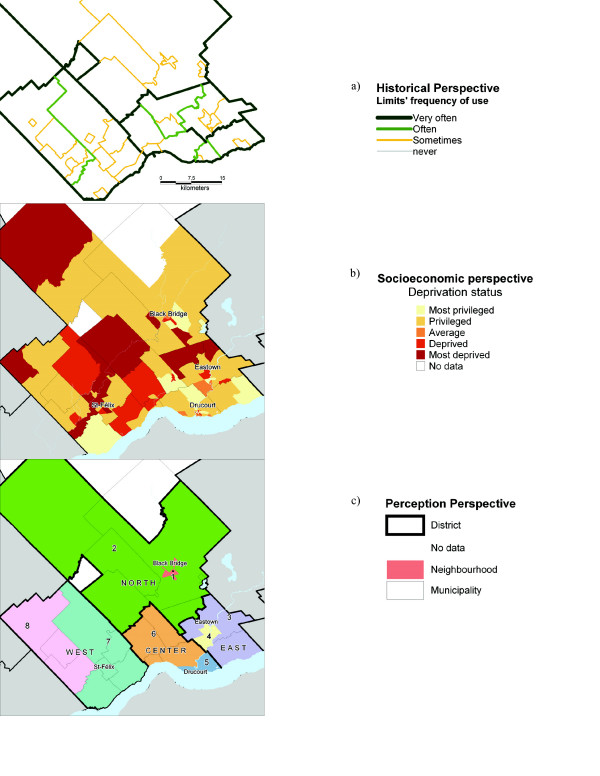
Verdier's neighbourhood definition from the historical^a^, socioeconomic^b ^and perception^c ^perspectives.

The use of the historical approach was particular in the case of Verdier. Since the road network is only seldom used as a border, it is mainly on the basis of municipal limits (ancient and recent limits) that the analytical framework (the GIS) was built. Thus, we located municipalities which maintain links regarding functional services (firemen, ambulances, *etc*.) and closer social bonds (pastorals units, schools, *etc*.). Those associations interestingly revealed some kind of "natural" grouping between municipalities. Indeed, some localities tend to share services and institutions with some close localities and not with others. Figure 4a presents where these associations are if one considers limits often used. The socioeconomic perspective was less obvious to interpret in Verdier than in the other two territories. The grouping's spatial adjacency was weak and no scheme of deprivation was observed (figure 4b). However, it did highlight the most urbanized sectors, Black-Bridge, Eastown, Drucourt and Saint-Félix, whose deprivation level clearly differed from the surrounding rural environment.

The final result showed a set of eight rural neighbourhood units (figure 4c). The construction of these units was mainly based on three major characteristics. Unlike Banville, Verdier has a strong spatial frame of reference. All local actors quickly agreed that neighbourhood units had to be contained by what is known as the West, the North, the Center and the East (figure 4c). Within these territories, units were defined according to natural associations between some close municipalities (e.g. units 7 and 8), and on the rural-urban opposition pattern (units 1, 4 and 5). Units 1 and 4, however, were not established following a consensus. While making note that they were not optimal, we kept these units because most participants agreed they best represent the urban-rural opposition while keeping an integer number of DAs. Other criteria were also considered by the local actors, in particular social contacts, economic poles' attraction, and the municipality's sense of belonging. One can note that in no case was the level of deprivation considered as being a significant criterion to define those rural neighbourhood units.

### Neighbourhood Unit's Profile

Preliminary analysis of various aspects such as socioeconomic indicators and measures of life and disability-free life expectancies had already shown major differences between neighbourhood units. We found major differences between neighbourhood units within and between the three territories.

Indeed, variations of almost $10,000 in annual personal income were observed between two neighbourhoods within the borough of Saint-Louis. In the same territory, we observed a difference of 23% between the proportion of the population made up of single-parent families between units 5 and 6, and of 24% similarly for people with no high school degree between units 6 and 9. Concerning life expectancy at birth (LE) in Saint-Louis, 5 of the eleven units differed significantly from Saint-Louis taken as a whole. Likewise, for disability-free life expectancy at birth (HE), 4 units differed significantly from Saint-Louis, while the gap between neighbourhoods (units 3 and 11) could reach as much as 16 years (Table 1), which is extremely high considering that these areas are only 600 meters apart.

**Table 1:** Population, socioeconomic context, life and heath expectancy in districts and neighbourhood of Saint-Louis, Banville and Verdier.

		**Population**			**Socioeconomic**				**Life expectancy**		**Disability-free life expectancy**	
		Total	Young^a^	Elderly^b^	Income^c^	Education^d^	Living^e ^Alone	Single^f ^parent family				
**Vieux-Saint-Louis**		n	%	%	$	%	%	%	LE^g^	CI^i^	DLE^h^	CI^i^
Lafontaine		15925	10.2	21.5	20090	33.4	27.0	24.5	79.0*	*(77.8;80.1)*	73.0*	*(71.6;74.4)*
	1	3125	11.7	17.0	17958	37.2	28.2	33.5	77.2	*(74.4;80.0)*	71.5	*(68.0;75.0)*
	2	5200	10.1	25.0	21148	35.7	25.5	21.6	78.9	*(76.6;81.3)*	72.8	*(70.1;75.6)*
	3	3215	8.9	22.2	23362	29.6	26.0	23.8	86. 1*	*(83.7;88.5)*	80.1*	*(77.6;82.7)*
	10	4385	10.1	20.2	17960	30.7	28.5	21.6	76.4	*(74.1:78.7)*	70.1	*(67.3;72.9)*

Iberville		14555	13.6	18.1	16999	43.1	25.5	28.7	76.9	*(75.7;78.2)*	70.0	*(68.6;71.4)*
	4	2485	9.9	14.5	14737	39.2	29.4	26.4	85.8*	*(80.1;91.5)*	72.9	*(66.2;79.5)*
	5	4360	12.2	17.8	19509	38.9	21.5	20.6	78.8	*(76.5;81.0)*	73.0	*(70.6;75.5)*
	6	2145	23.6	9.8	14101	50.8	15.9	44.1	78.1	*(74.9;81.3)*	68.6	*(65.0;72.1)*
	7	5565	12.6	23.4	17161	45.2	30.5	29.7	75.7	*(73.8;77.7)*	69.2	*(67.0;71.4)*

V.-St-Louis		14505	10.9	18.8	18588	35.4	28.0	32.3	75.5*	*(74.3;76.6)*	68.3	*(67.5;70.2)*
	8	4290	11.4	18.2	16159	38.0	26.8	34.2	74.7*	*(72.6;76.8)*	68.2	*(65.8:70.6)*
	9	6010	10.1	18.8	22153	27.0	25.7	28.7	80.8*	*(78.8;82.8)*	74.3*	*(72.1;76.6)*
	11	4205	11.5	19.2	15975	44.6	32.4	36.0	71.9*	*(69.5;74.3)*	64.1*	*(61.3;67.0)*

**Banville**												

A		10280	17.3	8.0	26536	26.1	7.4	16.4	80.6	*(79.1;82.0)*	73.7*	*(72.0;75.4)*
	1	5035	16.2	9.0	25804	26.8	7.7	14.6	78.1*	*(76.2;79.9)*	72.9*	*(70.8;75.0)*
	2	5245	18.3	7.1	27238	25.5	7.1	18.2	83.1	*(80.7;85.5)*	74.2	*(71.3;77.0)*

B		15850	15.7	10.9	24672	24.6	8.6	18.1	81.6	*(80.6;82.7)*	76.7	*(75.5;77.9)*
	3	6775	17.3	8.0	25600	19.7	6.9	16.3	83.2*	*(81.3;35.2)*	78.4*	*(76.2;80.6)*
	4	5155	13.8	14.4	23146	29.4	11.7	20.4	81.4	*(79.4;83.5)*	76.9	*(74.7;79.2)*
	5	3920	15.7	11.4	25080	26.9	7.5	17.8	80.9	*(79.0;82.7)*	74.5	*(72.4;76.6)*

C		8145	17.8	6.4	30883	18.3	10.1	17.9	81.7	*(80.1;83.3)*	76.5	*(74.4;78.5)*
	6	4255	15.0	7.8	32536	19.5	13.4	18.4	83.7	*(80.9;86.6)*	77.8	*(74.4;81.2)*
	7	3890	20.8	4.9	29075	17.0	6.6	17.4	82.9	*(80.4;85.3)*	76.8	*(73.5;80.0)*

D		15440	12.6	16.9	26614	21.3	14.0	15.3	81.3	*(80.0;82.5)*	77.4	*(75.9;79.0)*
	8	4495	13.0	15.7	27269	19.6	12.3	17.5	82.2	*(79.5;84.3)*	77.5	*(74.5;30.6)*
	10	6290	15.3	13.4	27295	20.6	7.1	11.6	81.3	*(79.0;83.7)*	78.0	*(75.0;81.0)*
	13	4655	8.4	13.9	25091	23.9	24.9	18.6	80.5	*(78.2;82.7)*	76.7	*(74.1;79.4)*

E		9210	12.9	19.3	27400	23.0	11.6	16.1	78.9*	*(77.5;80.4)*	74.8	*(73.0;76.6)*
	9	4905	12.9	15.4	28809	21.3	9.7	16.3	83.4	*(79.8;87.0)*	78.0	*(73.2;82.8)*
	11	4305	12.8	24.0	25795	25.1	13.8	15.6	77.9*	*(76.5;79.4)*	73.9*	*(72.3;75.5)*

F		11360	11.0	22.5	20638	35.3	24.0	22.9	81.6	*(80.1;83.2)*	74.7	*(73.0;76.5)*
	12	5765	11.9	22.9	20022	36.8	25.7	24.9	82.0	*(79.7;84.3)*	75.6	*(73.0;78.2)*
	14	2385	9.9	22.0	23064	29.2	20.1	24.1	81.7	*(77.2;86.2)*	73.3	*(67.8;78.8)*
	15	3210	10.3	22.1	19944	36.9	23.6	18.4	80.1	*(78.3;82.0)*	73.5*	*(71.5;75.4)*

**Verdier**												

North		11560	16.4	15.2	21875	41.7	12.6	10.3	79.3	*78.1;80.4*	75.0	*(73.7;76.3)*
	1	3235	12.4	23.5	21120	41.9	16.7	17.1	76.6	*73.9;73.2*	72.6	*(69.4;75.8)*
	2	8325	18.0	12.0	22177	41.6	10.9	7.6	80.3	*79.0;81.6*	75.5	*(74.1;77.0)*

East		10485	20.1	11.0	24798	28.3	8.5	12.6	82.9*	*81.6;84.2*	78.8*	*(77.3;80.4)*
	3	5510	19.2	9.3	24860	30.5	8.1	10.3	81.4*	*79.7;83.1*	77.4*	*(75.4;79.5)*
	4	4975	21.1	12.8	24735	26.0	8.9	15.3	83.0*	*81.1;85.0*	79.1*	*(76.9;81.3)*

Center		12855	15.7	16.7	23569	34.7	12.4	14.7	77.5*	*76.3;78.7*	73.2*	*(71.9;74.6)*
	5	6730	15.2	17.7	24919	32.5	12.7	14.4	78.5	*77.0;80.0*	73.8	*(72.3;75.4)*
	6	6125	16.3	15.7	22088	37.1	12.1	15.0	76.6*	*74.7;78.5*	72.7*	*(70.5;74.8)*

West		9645	15.3	20.5	20601	40.2	12.5	12.6	79.4	*78.1;80.7*	73.9	*(72.5;75.4)*
	7	6285	15.9	20.0	21136	40.0	11.0	14.8	81.0	*79.3;82.7*	75.7	*(73.7;77.7)*
	8	3360	14.3	21.6	19631	40.6	15.3	8.1	77.0	*74.8;79.2*	71.1*	*(68.8;73.5)*

a: Percentage of persons below age 15 in total population.

b: Percentage of persons over age 65 in total population.

c: Mean personal income of persons age 15 and over in Canadian $.

d: Percentage of persons with no degree, certificate or diploma in population of age 15 and over.

e: Percentage of persons living alone in population age 15 and over in private households.

f: Percentage of single parent families in all types of families.

g: Average number of years to live at birth.

h: Average number of years to live at birth without disability.

i: Confidence interval at 95%.

* : LE or DLE statiscally different from the region as a whole (0.05 > p)

**SOURCES**: Quebec death data base (1996–2002); Canadian census (2001).

Tables 2 and 3 illustrate the same types of differences for Verdier and Banville. The variations in life and disability-free life expectancies at birth are smaller inside both Verdier and Banville. However, with respect to the socioeconomic context, it was inside Banville and Saint-Louis that the greatest differences were observed, whereas in Verdier the socioeconomic differences between the units were often two times smaller than in the urban sectors. Thus, one can conclude that the neighbourhood units we constructed for Saint-Louis, Banville and Verdier present large differences between them in terms of socioeconomic characteristics and population health status. More disparities based on these neighbourhood units were observed and are reported elsewhere [48-50].

### Knowledge Exchange

The above results were presented in each territory between February and May 2007. Local stakeholders, including elected officials, participated in a three-hour session where socioeconomic and health disparities were presented and discussed on the basis of our neighbourhood units. On the whole, the results presented reinforced the intuitive knowledge stakeholders had of their territory. However, it appeared that the magnitude of health disparities between neighbourhoods was larger than expected by stakeholders and this raised questions and generated discussions about public policies and planning issues, such as favouring social mixing in new housing projects, developing public transit services in some remote rural neighbourhoods and reinforcing social networks among isolated people (living alone) and single-mother families. In the end, both researchers and local stakeholders agreed that this exchange of information proved to be an empowering experience for future collaboration in the same area.

## Discussion

This study is an implementation of a multi-perspective approach for defining neighbourhood units in three territories located in the Quebec City region. Our purpose was two-fold. First, we wanted to show how it is possible to integrate different ways of defining neighbourhood units and produce significant information on health variations at a local scale. Second, we sought to incorporate the point of view of local key actors in defining such units in order to facilitate the exchange of knowledge between researchers and local stakeholders. Results show that this exercise was feasible and successful. Three methodological approaches were integrated to delineate neighbourhood units in the selected territories. Huge socioeconomic and health variations were found between neighbourhood units and within territories, and these variations raised much interest among local stakeholders.

We have seen that in defining neighbourhood units, each perspective had its role or input in the process. The historical approach reveals boundaries usually inherited from the institutional framework which significantly cut out the territory, and to evaluate the presence, the strength, or the coherence of the spatial frame of reference. The socioeconomic approach rather highlights the actual homogeneity or heterogeneity of a territory's deprivation and its spatial distribution. Provided with these two geographical representations, local actors can determine which elements of these perspectives, or other elements drawn from their own experience, are most significant in defining neighbourhood units.

We consider this whole process fundamental to render the concept of neighbourhood operational, since it uses an objective methodology that integrates elements of subjectivity and reflects the singularity of the territory. Indeed, inner characteristics identified by actors were aspects which could only be pointed out by people having an intimate experience of the territory. As Suttles [25] and Galster [22]note, what distinguishes one neighbourhood from another is the way various specific elements combine among themselves, thereby conferring its idiosyncrasy to the neighbourhood.

The scale at which neighbourhood units were constructed was mainly determined by two features: the historical boundaries that qualify the general spatial frame of reference, especially in rural environments, and the statistical constraint that keeps neighbourhood populations in the range of 2,000 to 8,000. The latter, however, has mainly influenced bordering DAs of each unit, which were dispatched according to the workshop's participants.

Even though the spatial frame of reference was very different for the urban boroughs of Banville and Saint-Louis, it is interesting to note that criteria evoked by local key actors during the workshops were often the same for both places. Indeed, main roads or railways, housing types, the general level of deprivation, the presence of social contacts and several historical boundaries were all important elements used as guideposts by the workshop participants. Thus, it was possible to integrate the historical and socioeconomic perspectives by having resorted to the local actors' personal knowledge. Indeed, results showed an efficient way to present general health outcomes at the local scale that could be easily understood by local stakeholders, while being compatible with national and provincial health databases. Each one of these units had its own characteristics; however, it should be known that no characteristic was used as a global beacon for a unit's definition. Consequently, local actors individually bounded each one of these units according to one or several of these indicators that specifically characterized them.

Nevertheless, our work was not carried out without difficulty. The constraint of using an integer number of DAs to delimit units was very cumbersome for local key actors, and had the effect of reducing the precision of the "natural" groupings suggested. Moreover, keeping a unit's population within an interval of 2,000 to 8,000 individuals was sometimes awkward for workshop participants. Actually, a few sub-sectors having approximately 1,000 inhabitants should have been isolated for a more accurate representation of their territory's perception. However, in the context of health studies carried out at a local level and for reasons of statistical precision, it was not possible to reduce the size of neighbourhood units. When very small populations are involved, perhaps other types of analyses, more qualitative in nature, would be more appropriate.

In a mostly rural area like Verdier, the same kind of problems arose but with greater impact on the significance of the final set of units, and for several reasons. First, it was harder to find homogeneity or some socioeconomic similarity in this area because of the large expanse of the area and the low population density. We also noted from discussants' comments that the rural area, at the local scale, was perceived to have a level of heterogeneity that is seldom observed in an urban area, and that the use of DAs could not help to recognize. Second, indicators mentioned by Verdier's workshop participants differed in nature from those mentioned for the urban boroughs. Those indicators reflected indeed a very different social reality and were adapted according to the scale at which space is used by the inhabitants. Whereas boroughs of Banville and Saint-Louis are in fact only part of a much larger functional whole (Quebec City), the regional county of Verdier is formed by 18 municipalities, each enjoying a relatively good deal of autonomy. Consequently, the neighbourhood concept took a new dimension here and often extended to bordering municipalities with which social or administrative contacts were more frequent and common.

In the literature, the concept of neighbourhood is rarely used in relation to the countryside [32]and defining neighbourhood units in rural areas now represents an important methodological challenge [12]. Based on our study, we can suggest that rural neighbourhood units are entities which share many institutions and public services and are often linked by an economic pole. It is mainly through these elements that our rural neighbourhood units were created.

The quality of our final set of neighbourhood units, in urban or rural areas, can be closely related to the quality and diversity of the local key actors who took part in these workshops. However, the results' significant value comes precisely from the fact that, in spite of their various expertises, they succeeded in reaching a **consensus **on the neighbourhoods' representations. This procedure strengthened the final divisions, which we believe would not have been significantly different had we worked with other local key actors, especially in urban areas. In fact, many boundaries were selected without much discussion as they were already known by all participants.

Different ways of cutting up territories could lead to different results in terms of people's health; in other words, this type of exercise brings up the modifiable areal unit problem. According to Openshaw [43], the MAUP is created by the incertitude by which a set of geographical units should be used. The method presented in this article provides a set of geographical units based on a consensus made by local actors. With this consensus, we believe their spatial distribution can be used to qualify their idiosyncrasy and be less vulnerable to the MAUP.

Also, as our final set of neighbourhood units was not compared with more conventional ones, based exclusively on socioeconomic indicators, for instance, we do not know if this set brings greater geographical health disparities and provides more useful insights into the determinants of health inequalities. First, recall that our set of units found huge health inequalities, up to 16 years of health expectancy in Saint-Louis. Second, let us add that, in a recent study [49], the same set of units was used successfully in depicting variations in perception of place (problems and social cohesion in the neighbourhood) and their impact on people's health. Different or similar results could have been obtained with more conventional geographical divisions and, in future work, we will check for this. Be that as it may, the set of units proposed in this paper will always be the one preferred by local stakeholders, since it is a reflection of their own perceptions of place and of the ways they work on a day-to-day basis in their milieu.

In our opinion, the present study makes an original and important contribution to the field of research on neighbourhood and health. Fist, it gives substance to an ambiguous and vague concept. Second, it exemplifies a three-fold approach for defining neighbourhood units that goes beyond the usual socioeconomic criteria and administrative statistical units. Third, it considers not only cities but also rural areas, which are usually ignored in such exercises. Fourth, it shows how fruitful links can be created with local stakeholders and knowledge exchange facilitated. Finally, it proposes an approach which is reproducible elsewhere, in industrialized countries, despite differences in health information systems and local decision makers.

## Competing interests

All author(s) declare that they have no competing interest.

## Authors' contributions

AL is the principal investigator of this study. He has done the historical research, created the GIS, performed the statistical analysis, managed all discussion sessions, and wrote the article. RP and PYV supervised closely the entire work, on the conceptual and technical aspects, and substantially revised this manuscript many times. All authors read and approved the final manuscript.
